# Acute Acalculous Cholecystitis due to Viral Hepatitis A

**DOI:** 10.1155/2013/407182

**Published:** 2013-09-10

**Authors:** Safak Kaya, Ahmet Emre Eskazan, Nurettin Ay, Birol Baysal, Mehmet Veysi Bahadir, Arzu Onur, Recai Duymus

**Affiliations:** ^1^Department of Infectious Disease, Diyarbakir Training and Research Hospital, 21000 Diyarbakir, Turkey; ^2^Department of Hematology, Diyarbakir Training and Research Hospital, 21000 Diyarbakir, Turkey; ^3^Department of General Surgery, Diyarbakir Training and Research Hospital, 21000 Diyarbakir, Turkey; ^4^Department of Gastroenterology, Faculty of Medicine, Bezmialem University, 34000 Istanbul, Turkey; ^5^Department of General Surgery, Faculty of Medicine, Dicle University, 21000 Diyarbakir, Turkey; ^6^Department of Microbiology, Diyarbakir Training and Research Hospital, 21000 Diyarbakir, Turkey; ^7^Department of Radiology, Diyarbakir Training and Research Hospital, 21000 Diyarbakir, Turkey

## Abstract

Inflammation of the gallbladder without evidence of calculi is known as acute acalculous cholecystitis (AAC). AAC is frequently associated with gangrene, perforation, and empyema. Due to these associated complications, AAC can be associated with high morbidity and mortality. Medical or surgical treatments can be chosen according to the general condition of the patient, underlying disease and agent. Particularly in acute acalculous cholecystitis cases, early diagnosis and early medical treatment have a positive effect on the patient and protect them from surgical trauma. ACC is a rare complication of acute viral hepatitis A. Herein, we present an adult patient of acalculous cholecystitis due to acute viral hepatitis A. She responded to the conservative management.

## 1. Introduction

Hepatitis A is generally an acute, self-limited infection of the liver by an enterically transmitted hepatitis A virus (HAV). Infection may be asymptomatic or result in acute hepatitis, and rarely, fulminant hepatitis can ensue. Recognized complications of hepatitis A include cholestasis, prolonged and relapsing disease, fulminant hepatitis, and triggering of chronic active autoimmune extrahepatic disease [1]. Acute acalculous cholecystitis (ACC) is a rare complication of acute viral hepatitis [2]. Although the origin is obscure, demonstrated invasion of the gallbladder and bile duct epithelium by HAV and cell-mediated immunologic response have been proposed in the pathogenesis of HAV infection induced cholecystitis [3, 4]. Herein, we report a case of acalculous cholecystitis due to acute viral hepatitis A along with the published literature.

## 2. Case Report 

A 31-year-old female patient was admitted to the hematology department because of pancytopenia in February 2013. She had nausea, loss of appetite, back and joint pain, darkening of urine, and abdominal pain for 10 days. Her medical history was unremarkable. There was no history of medication or drug abuse. Physical examination showed body temperature of 37.5°C, heart rate of 92/minute, and blood pressure of 110/60 mmHg. Scleral icterus was present. The right side of the abdomen was tender with painful fullness in the right hypochondrium (a positive Murphy's sign). Her liver was painful and palpable 3 cm under right costal margin. After examination, she was referred to general surgery with a diagnosis of acute cholecystitis, and surgery was planned. Laboratory investigations revealed microcytic anemia, leukopenia, and thrombocytopenia, elevated levels of alanine aminotransferase (ALT), aspartate aminotransferase (AST), alkaline phosphatase (ALP), gamma glutamyltransferase (GGT), and total serum bilirubin (Table 1). She had iron deficiency anemia (reduced levels of serum iron and serum ferritin with increased total iron binding capacity (TIBC)). Prothrombin time, C-reactive protein, erythrocyte sedimentation rate, and urinalysis were normal. Brucella standard tube agglutination (STA) test was negative. Abdominal ultrasound revealed hydropic gallbladder without calculus, thickened gallbladder wall (14 mm), perivesical liquid accumulation, and hepatosplenomegaly. Serology for viral hepatitis suggested acute hepatitis A infection (anti-HAV IgM (+) and anti-HAV IgG (+)) and was negative for other causes (HBsAg (−), anti-HBcIgM (−), and anti-HCV (−)). The diagnosis was acute viral acalculous cholecystitis. Patient was treated with supportive therapy of intravenous (I.V.) fluid, I.V. metoclopramide, and I.V. ranitidin with low fat and high carbohydrate diet. Her symptoms regressed within four days, and the biochemical markers including serum ALT, AST, and total bilirubin levels decreased (Table 1). The gallbladder wall thickness was regressed to 3.5 mm, and no surgical intervention was required. She was discharged on the 8th day of admission. During the followup, she was in good condition without any complaints with improved biochemical tests in March 2013.

## 3. Discussion

Inflammation of the gallbladder without evidence of calculi is known as ACC [5]. AAC is frequently associated with gangrene, perforation, and empyema. Due to these associated complications, AAC can be associated with high morbidity and mortality [6]. The pathophysiology of the acalculous cholecystitis during acute viral hepatitis is not clear: hypoalbuminemia, local extension of the hepatic inflammatory process, and elevated portal pressure all could be reflected as the edema of the gallbladder wall [7]. The diagnosis is suspected clinically and then confirmed through ultrasound. The ultrasonographic criteria for diagnosing AAC include (1) gallbladder distention; (2) thickening of the gallbladder wall (>3.5 mm); (3) no acoustic shadow or biliary sludge; (4) perivesical liquid accumulation; and (5) no dilatation of the intra- and extrahepatic bile ducts. The sensitivity of ultrasound for detection of AAC is 88.9%, and the specificity and accuracy are 97.8 and 96.1%, respectively. Treatment is initially conservative, with indications for urgent cholecystectomy in cases of gangrene or perforation of the gallbladder wall [8]. HAV induced AAC has been very rarely reported. Indeed, by searching the MEDLINE database for published articles using the words “acalculous cholecystitis” and “viral hepatitis A,” we identified only 20 reports in the literature, including twenty-two patients with ACC due to acute viral hepatitis A [2, 3, 8–24] of which fifteen had appropriate information for analysis (Table 2). Four of these publications were in adults [9, 11, 13, 22].

Ozaras et al. [11] described two adult patients (28 and 20 years of age) with acute cholecystitis due to HAV infection. Both patients had acute HAV infection documented by biochemical, serologic, and clinical features. Cholecystitis developed during the course of the disease but did not lead to an acute phase response and required neither administration of antibiotics nor surgical intervention. With a close followup, both of the patients had fully recovered. Melero Ferrer and coworkers [14] reported a 39-year-old woman with fever, abdominal pain, and moderately elevated transaminase levels who developed jaundice and peritoneal irritation. Diagnosis of acute cholecystitis was given by abdominal ultrasound and magnetic resonance imaging. The patient underwent surgery. In the postoperative period, positive IgM antibody titers for HAV were obtained, confirming the diagnosis of HAV infection. Black and colleagues [2] reported a 6-year-old child presenting with gangrenous cholecystitis due to HAV infection. Ultrasonography showed a slightly distended gallbladder containing echogenic bile. Laparotomy revealed a distended gallbladder with areas of necrosis. Dalgic et al. [12] presented case had acute HAV infection with acalculous cholecystitis developed during the course of the disease. Surgical intervention was not required in their patient. A repeated imaging with ultrasonographic findings regressed after 4 days of admission. Mourani et al. [9] described a patient with acute cholecystitis due to HAV infection. They detected the HAV antigen immunohistochemically in the gallbladder which was removed by laparoscopy. This patient was operated on considering a diagnosis of cholecystitis with ascending cholangitis. Both ultrasonography and retrograde cholangiopancreatogram showed thickening of the gallbladder wall. 

Acute hepatitis A virus (HAV) infection is frequently encountered in developing countries especially in children [11]. In our country, anti-HAV IgG positive rate in adult patients is between 85 and 100% in different studies [25]. Our case is an adult patient. Looking at the literature, majority of the cases are children. Hepatitis A virus infection should be considered as a cause of acute acalculous cholecystitis in adult patients, in countries in which the disease is mainly passed in childhood such as our country. 

The treatment of AAC varies depending on the clinical presentation. Most cases are self-limited, and the gallbladder may spontaneously decompress with treatment of the underlying systemic disease within approximately two weeks. Associated complications such as gallbladder perforation and deterioration of abdominal signs have been suggested as indications for surgery [16]. Eleven of fifteen patients that had appropriate information for analysis in previous reports were managed conservatively [8, 11–13, 15, 16, 18–20] and others with surgical intervention [2, 9, 10, 14].

The case reported here is an adult patient who presented with HAV, which was confirmed serologically, and symptoms suggestive of acute cholecystitis with pancytopenia. Ultrasonographic examination revealed the diagnosis of acalculous cholecystitis which required neither antibiotic treatment nor surgical intervention. The anemia was due to iron deficiency which improved with oral iron supplements, and leukopenia and thrombocytopenia were normalized during the followup (Table 1). 

In conclusion, however, ACC is an extremely rare complication of acute viral hepatitis A, and mortality from ACC in patients with viral hepatitis A is extremely low in comparison to ACC of other origins that need urgent surgical intervention. It should be kept in mind that acute viral cholecystitis can develop during the course of acute HAV infection. Hence, conservative therapy may be adequate, so we can avoid unnecessarily invasive procedures.

## Figures and Tables

**Table 1:** Laboratory findings of the patient during the follow-up period (ALP: alkaline phosphatase; ALT: alanine transaminase; AST: aspartate transaminase; GGT: gamma-glutamyltransferase; Hb: hemoglobin; WBC: white blood cells).

Day(s)	Parameter								
	WBC (4.1–11.2 × 10^9^/L)	Hb (12.5–16 gr/dL)	Platelet (150–400 × 10^9^/L)	ALT (0–41 U/L)	AST (0–40 U/L)	GGT (5–61 U/L)	ALP (<240 U/L)	Total bilirubin (0–1.2 mg/dL)	Direct bilirubin (0–0.3 mg/dL)
1st	3.3	9.5	139	618	559	147	371	2.11	1.92
4th	2.2	8.3	138	533	360	120	148	1.45	0.92
7th	3.0	8.9	190	247	88	172	269	1.19	0.53
20th	4.4	10.6	275	36	27	57	196	0.63	0.2

**Table 2:** Review of the clinical presentation, ultrasound findings with treatment modalities, and outcomes of patients with acalculous cholecystitis due to viral hepatitis A published in the literature (F: female; M: male; NA: not available; USG: ultrasonography).

Publication, author, and year, (Ref.)	Cases, *n*	Age, year/sex	Clinical presentation	USG	Treatment	Follow-up time, months	Outcome
Black and Mann 1992 [2]	1	6/M	NA	Distended gallbladder	Surgery	NA	NA
Mourani et al. 1994 [9]	1	68/M	Fever, nausea, vomiting, and chills	Thickened gallbladder wall	Surgery	1	Cure
Ciftci et al. 2001 [10]	1	7/M	Abdominal pain, distention, icterus, and fatique	Subhepatic fluid and thickened gallbladder wall (14 mm)	Surgery	6	Cure
Ozaras et al. 2003 [11]	2	28/M	Malaise, abdominal pain, dark urine, and anorexia	Thickened gallbladder wall	Conservative therapy	3	Cure
		20/F	Jaundice, nausea, vomiting, malaise, pruritus, and anorexia	Hepatosplenomegaly and a hydropic gallbladder without calculus	Conservative therapy	6	Cure
Dalgic et al. 2005 [12]	1	11/F	Fever, fatigue, nausea, vomiting, abdominal pain, and loss of appetite	Hydropic gallbladder without calculus, thickened gallbladder wall (12 mm), and pericholecystic fluid	Conservative therapy	6	Cure
Kayabas et al. 2007 [13]	1	15/M	Fever, nausea and vomiting, abdominal pain, loss of appetite, dark urine, and pale stool	Thickened gallbladder wall	Conservative therapy	1	Cure
Melero Ferrer et al. 2008 [14]	1	39/F	Fever, abdominal pain, and jaundice	Thickened gallbladder wall (7.1 mm)	Surgery	1	Cure
de Souza et al. 2009 [8]	1	16/M	Abdominal pain, fever, nausea, vomiting, and cephalalgia	Thickened gallbladder wall (7.0 mm)	Conservative therapy	NA	NA
Arroud et al. 2009 [15]	1	11/E	Fever, asthenia, vomiting, and abdominal pain	Thickened gallbladder wall (11 mm)	Conservative therapy	1	Cure
Suresh et al. 2009 [16]	1	2.5/F	Fever, nausea and vomiting, abdominal pain, loss of appetite, dark urine, and pale stool	Hepatosplenomegaly, hydropic gallbladder without calculus, thickened gallbladder wall, and pericholecystic fluid	Conservative therapy	NA	NA
Arcana et al. 2011 [17]	1	14/M	Abdominal pain, nausea, fever, and jaundice	Hepatomegaly and thickened gallbladder wall (7.0 mm)	NA	NA	NA
Hasosah et al. 2011 [18]	1	13/F	Fever, vomiting, and jaundice	Thickened gallbladder wall	Conservative therapy	NA	Cure
Herek et al. 2011 [19]	1	9/M	Nausea, vomiting, fever, and abdominal pain	Gallbladder wall thickening (9.7 mm) and pericholecystic-free fluid	Conservative therapy	6	Cure
Prashanth et al. 2012 [20]	1	12/F	Abdominal pain and vomiting	Gallbladder wall edema and echogenic biliary sludge	Conservative therapy	6	Cure
